# GarlicESTdb: an online database and mining tool for garlic EST sequences

**DOI:** 10.1186/1471-2229-9-61

**Published:** 2009-05-18

**Authors:** Dae-Won Kim, Tae-Sung Jung, Seong-Hyeuk Nam, Hyuk-Ryul Kwon, Aeri Kim, Sung-Hwa Chae, Sang-Haeng Choi, Dong-Wook Kim, Ryong Nam Kim, Hong-Seog Park

**Affiliations:** 1 Genome Research Center (GRC), Korea Research Institute of Bioscience and Biotechnology, Daejeon 305-306, Korea; 2 University of Science and Technology (UST), Daejeon 303-333, Korea; 3 Gnc Bio Co Ltd, Daejeon 305-801, Korea; 4 Mokpo National University, Jeonnam 534-729, Korea

## Abstract

**Background:**

*Allium sativum*., commonly known as garlic, is a species in the onion genus (*Allium*), which is a large and diverse one containing over 1,250 species. Its close relatives include chives, onion, leek and shallot. Garlic has been used throughout recorded history for culinary, medicinal use and health benefits. Currently, the interest in garlic is highly increasing due to nutritional and pharmaceutical value including high blood pressure and cholesterol, atherosclerosis and cancer. For all that, there are no comprehensive databases available for Expressed Sequence Tags(EST) of garlic for gene discovery and future efforts of genome annotation. That is why we developed a new garlic database and applications to enable comprehensive analysis of garlic gene expression.

**Description:**

GarlicESTdb is an integrated database and mining tool for large-scale garlic (*Allium sativum*) EST sequencing. A total of 21,595 ESTs collected from an in-house cDNA library were used to construct the database. The analysis pipeline is an automated system written in JAVA and consists of the following components: automatic preprocessing of EST reads, assembly of raw sequences, annotation of the assembled sequences, storage of the analyzed information into MySQL databases, and graphic display of all processed data. A web application was implemented with the latest J2EE (Java 2 Platform Enterprise Edition) software technology (JSP/EJB/JavaServlet) for browsing and querying the database, for creation of dynamic web pages on the client side, and for mapping annotated enzymes to KEGG pathways, the AJAX framework was also used partially. The online resources, such as putative annotation, single nucleotide polymorphisms (SNP) and tandem repeat data sets, can be searched by text, explored on the website, searched using BLAST, and downloaded. To archive more significant BLAST results, a curation system was introduced with which biologists can easily edit best-hit annotation information for others to view. The GarlicESTdb web application is freely available at .

**Conclusion:**

GarlicESTdb is the first incorporated online information database of EST sequences isolated from garlic that can be freely accessed and downloaded. It has many useful features for interactive mining of EST contigs and datasets from each library, including curation of annotated information, expression profiling, information retrieval, and summary of statistics of functional annotation. Consequently, the development of GarlicESTdb will provide a crucial contribution to biologists for data-mining and more efficient experimental studies.

## Background

EST sequencing is a useful tool for investigating a wide variety of genetic characteristics of a species, such as how many genes exist in the species, how gene expression patterns differ between tissues, where exonic and intronic regions are located and how many alternatively spliced transcripts can be created from a single gene [1-3]. In addition, ESTs have proven to be extremely useful for making DNA-based molecular markers, including microsatellites, SSRs (simple sequence repeats) and SNPs (single nucleotide polymorphisms) [4,5]. In this respect, EST information is regarded as a valuable resource for evaluating genetic diversity, for marker-assisted breeding, and for capturing and mining the transcript of a gene, to predict its protein product and eventually its function.

With steady advances in high-throughput sequencing technology, a number of researchers have attempted to obtain a more abundant EST data set, creating a desire to manipulate huge quantities of EST data more quickly and accurately to keep up with the pace of data generation. Efforts to analyze ESTs have employed various strategies such as: ESTIMA, ESTAnnotator, and ESTminer as web applications and database schema [6-8]. Until very recently, however, there was no an integrated database or tools available to curate finished annotations and simultaneously provide processed EST information about garlic (*Allium sativum*).

For this reason, we developed the GarlicESTdb using a pipeline system. The GarlicESTdb provides a handy way to access all the available garlic EST resources and be integrated with comprehensive information, which includes information about cluster, annotation, protein domain, pathway, tandem repeat, SNP, and so on. In addition, the GarlicESTdb makes it possible to manage annotation data in a manner that can be readily changed according to personal inclination, as well as to access abundant and valuable EST information.

## Construction and content

### Database overview

GarlicESTdb has been developed on a platform comprised of the Apache/Tomcat web server on RedHat9.0 and uses the MySQL5.0 database management system. The web application was implemented with JSP (Java Server Pages)/EJB (Enterprise Java Beans)/JavaServlet technology and AJAX framework. GarlicESTdb provides users with the flexibility to perform all analysis steps separately. In addition, all the information in each project library can be accessed and searched through the web application.

**Figure 1:**
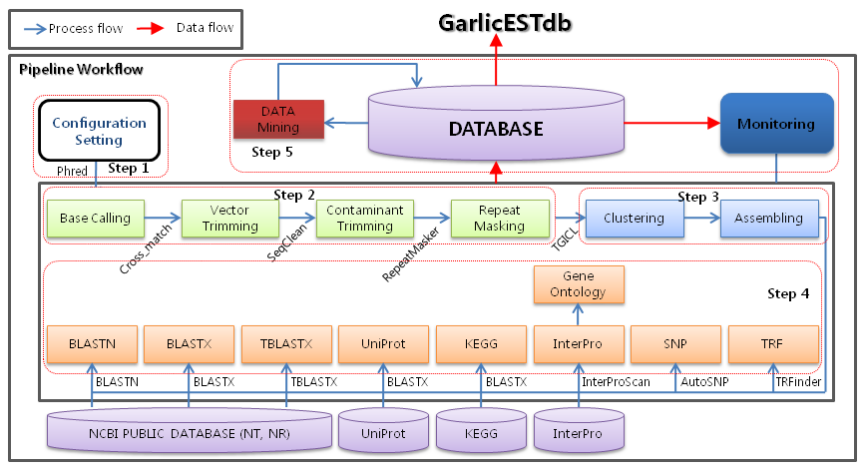
**Workflow schema of the pipeline**. The pipeline consists of five steps within the red-dotted rectangle: configuration, cleansing, clustering and assembling, annotation, and data mining & visualization.

In the present work, a total of 21,595 garlic EST sequences derived from four cDNA libraries made from leaf and stem tissues of Korean and Chinese garlic plants were used to construct the database (refer to the "cDNA library construction" on the website for details). To analyze these data, we have built a pipeline using the JAVA programming language as shown in Figure 1. The pipeline is composed of a series of fully integrated systems and will automatically process, analyze and store the data into a MySQL database management system. Major modules of the analysis pipeline include the following programs:

(i) Pre-processing (Step 1, 2 and 3): a bundle of background pipelines for cleaning raw DNA sequences, clustering reads and assembling contigs.

(ii) Functional annotation (Step 4): a set of programs for annotation and storage of results in a relational database using various tools.

(iii) Data mining & Visualization (Step 5): a web application for providing higher-level information on processed sequence status and functional annotation, and for curating the biological meaning assigned to all EST consensus sequences (for more detailed information about the programs and parameters used in each step, please refer to "User Guide" at the top of the main page on the GarlicESTdb site).

The database contains:

(i) Pre-processed data including trace data, cleaned sequence reads, contigs, clustering and assembling data.

(ii) Functional annotation data from BLASTN, BLASTX, TBLASTX, UNIPROT, KEGG, INTERPRO, GO, TRF, and SNP.

(iii) Secondary mined data from electronic northern expression with annotated information gained from BLAST searches, and pathway information mapped using Basic, *E. coli *(*Escherichia coli*) and yeast pathways from KEGG.

## Utility and discussion

### Pre-processing

A typical EST sequence is only a very short copy of the mRNA itself and EST sequencing is highly error prone, especially at the ends. Contaminants should be removed before the sequences are used, to improve the efficacy of subsequent analyses. The pre-processing step can be divided into cleansing, clustering and assembling.

The cleansing is a basic part of processing used to obtain high-quality sequences from a raw EST dataset. After base calling performed with by Phred [9], the cleansing process executes Cross_match (version 0.990329), SeqClean , RepeatMasker (Smit *et al*., unpublished data and ) including a well-maintained all species repeat library by Repbase  continuously [10]. The cross_match program is used to identify and mask vector (pBK-CMV) and contaminant sequences, such as *E. coli*, chloroplast and mitochondria sequences. Our pre-processing pipeline runs cross_match twice, first to mask adaptor and then to mask vector sequences. After the cross_match finishes, SeqClean removes undetermined bases, poly (A) tails and low complexity regions. Repetitive elements, such as LINEs (Long interspersed elements), SINEs (Short interspersed nuclear elements) and LTRs (Long terminal repeats), can lead to erroneous assembly of sequences. Therefore, they are masked during the analysis using the RepeatMasker, which screens DNA sequences for interspersed repeats. The high-quality EST sequences and information about their cleansing as well as user-inputted raw EST sequences are stored in the database.

The clustering step is performed to collect overlapping EST sequences from the same transcript of a single gene into a unique cluster to reduce redundancy. The assembly step is performed to align and merge many fragments of a much longer DNA sequence in order to reconstruct the original sequence. For clustering and assembling, we use the TGICL and the CAP3 sequence assembly program that automates clustering and assembly of a large EST dataset [11,12]. The contigs and singletons that result from assembly are subjected to the annotation process.

After the pre-processing, all the results are stored in a database for evaluation and reporting, and the GarlicESTdb provides some tools and reports about pre-processing such as 'Reads Information', 'Trace Viewer', 'Contigs Information' and 'Contig Viewer'.

### Functional annotation

After the pre-processing step finished making EST consensus sequences, we assigned putative functions to the garlic ESTs based on BLASTN (Query Coverage ≥ 80.0%, Identity ≥ 70.0%, E-value ≤ 1.0E-20), BLASTX and TBLASTX (Match No. ≥ 50 aa, Identity ≥ 25.0%, E-value ≤ 1.0E-20) searches against the GenBank NT and NR databases  using a TimeLogic DeCypher system (Active Motif, Inc., ). The results were then parsed and stored in the GarlicESTdb. Metabolic pathway information is extremely important for applications involving the production of particular chemicals from garlic. To identify all metabolic genes and map them into KEGG pathway, supplementary annotations were created against the KEGG database  and the UniProtKB database  using BLASTX (Match No. ≥ 50 aa, Identity ≥ 25.0%, E-value ≤ 1.0E-20), and we use the InterProScan database to search for the functional motif (E-value ≤ 1.0E-4).

In order to gain a better understanding of the biological meaning of the ESTs, analysis of gene function was performed using GO terms provided by InterProScan results. The three categories (Biological Process, Cellular Component, and Molecular Function) were selected from the ontology hierarchy and referred to by GO id. Each GO id was linked to an AmiGO . As additional analysis tools, we used the Tandem Repeats Finder (TRF) to detect a copy number variants, and the AutoSNP to detect single nucleotide polymorphisms (SNPs) and insertion and deletion polymorphisms (INDELs) [13,14].

### Reporting, searching and secondary mining

**Figure 2:**
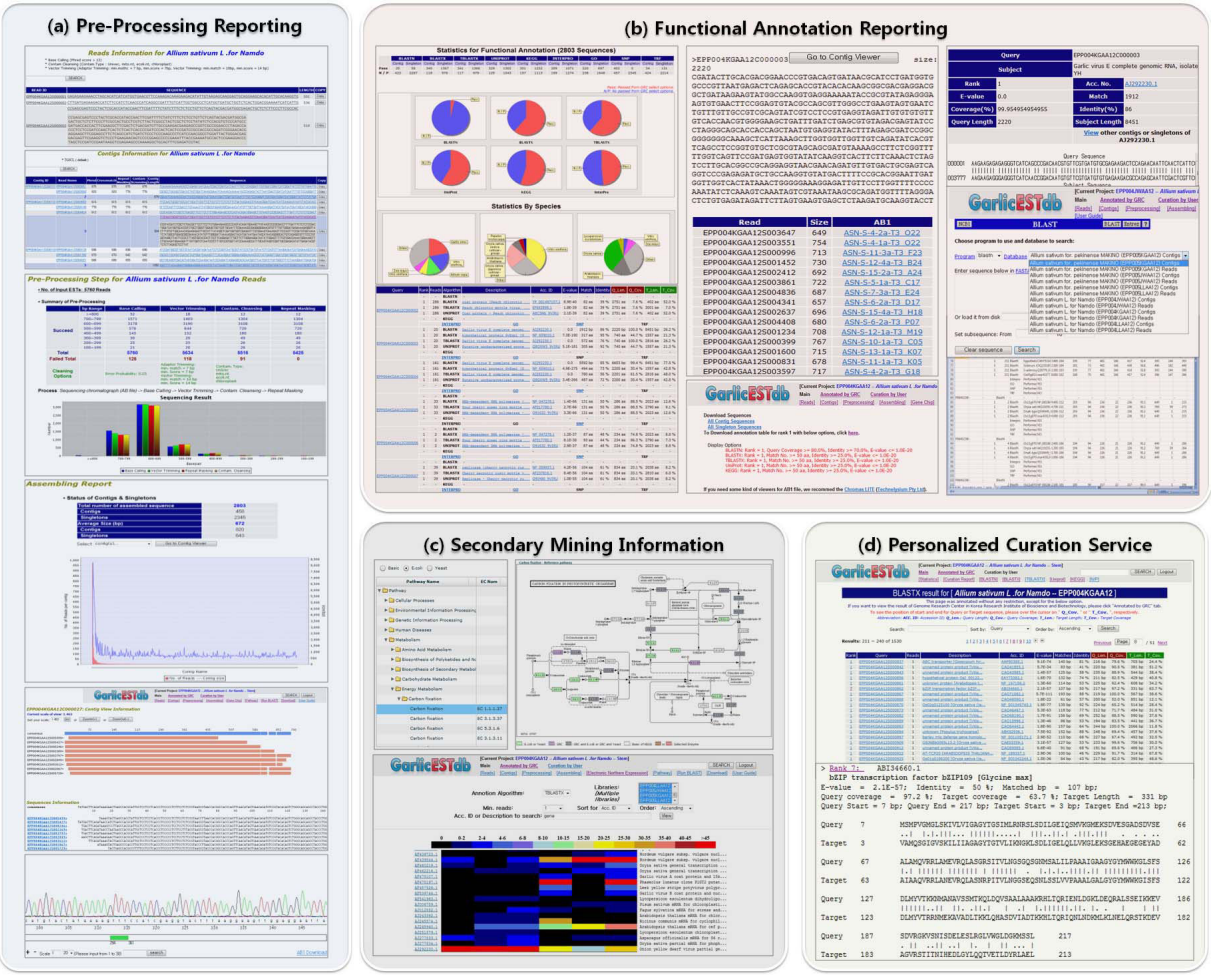
**Snapshots of the GarlicESTdb web application**. The panels show examples resulting from general functions. (a) An example of a pre-processing report showing cleaning sequences, assembly status, contig view and trace view. (b) An example of functional annotation reporting showings annotation statistics, annotation report, each annotated report (BLASTN, BLASTX, TBLASTX, etc), detailed information of consensus sequence, results of a BLAST search, data downloaded AB1 (contigs, singleton) and Excel. (c) An example of secondary mining information showing electronic northern expression information for all of garlic libraries and pathway information with enzymes annotations mapped from KEGG pathways. (d) An example of the personalized curation service. After free registration, users can modify the annotated results themselves. Please refer to the user's manual for details on the GarlicESTdb webpage.

We provide a web application to access the GarlicESTdb as shown in Figure 2. At first, the user can examine the pre-processing reports to evaluate the pre-processing pipeline from "Reads report", Contigs report", "Preprocessing report" and "Assembly report" (Figure 2a). Detailed functional annotation statistics for a cDNA library are provided in the 'statistics' link on the GarlicESTdb webpage. The preprocessing and assembly report provides a comprehensive summary of pre-processing information such as sequencing status, clustering information (in the contig viewer) and annotation result from each database. We also provide a trace viewer that allows users to evaluate the sequencing result on the web.

In addition to various reporting, the online database of GarlicESTdb provides a comprehensive information system for analyzing data from the garlic EST sequencing project, allowing users easy monitoring of all of analyzed EST datasets (Figure 2b). Existing information for all genes in an EST set can be retrieved by using full-text matching (against read id, consensus id, gene name, gene accession number, functional description and e-value score) from the search tool. This is particularly valuable for functional analyses, such as finding annotated EST consensus sequences or classifying genes according to functional descriptions.

Moreover, the GarlicESTdb provides access to the pathway information in KEGG. We mined the enzyme information from the annotated results of KEGG and UniProtKB database that contained an EC number in description (Figure 2c). This is useful for mapping the relationships among a whole system of annotated enzymes and is especially valuable for those who are interested in biological pathways. To aid in the analysis of expression patterns, we included the electronic northern expression module to detect the gene expression differences in each library through comparison with accession numbers from BLAST functional annotation (Figure 2c). It was colored according to the number of reads comprising its contigs. A newly identified expression profile might help to predict how expression might vary according to tissue and environment.

### Personalized curation service

In general, when EST consensus sequences are annotated, automated high-throughput EST analysis pipelines use BLAST to search for similar sequences in various relevant databases and then assign a putative function based on the description of best hits. At this time, for some genes in a species that have not been well studied before, the best hits contain many uninformative descriptions such as "hypothetical transcript" or "hypothetical protein". Thus, in order to make sense of the BLAST results and to offer intuitive annotation information, we developed a personalized curation service in which users can customize annotation results for further research (Figure 2d). In other words, individual users can change their best-hit results based on descriptions or using different thresholds for well-annotated BLAST output. This service improves the usability of the GarlicESTdb and provides foundations for more efficient experimental research on garlic.

### Genes involved in alliin biosynthesis and its breakdown

Our Garlic EST database contains EST sequences which are corresponding to cysteine synthases (CSase) and alliinases known to be key enzymes in alliin biosynthesis and the conversion of alliin to alliicin and its derivatives. In fact, the great interest and significant value of garlic in culinary, pharmaceutical and medicinal aspects and the source of the very characteristic garlic odor are likely to be originated from alliin biosynthesis and its breakdown in vivo [15,16]. We have found out three kinds of cysteine synthase, such as chloroplast cysteine synthase GCS2, putative S-allyl cysteine synthase and cysteine synthase from our garlic EST database, which had been constructed from leaf and stem tissues of Korean and Chinese garlic species, respectively (Additional file 1). Also, this garlic EST database contained three kinds of alliinase, such as alliin lyase 1 precursor (alliinase-1 or cysteine sulphoxide lyase 1), alliin lyase 2 precursor (alliinase-2 or cysteine sulphoxide lyase 2) and alliinase. In particular, we have found out only alliinase, but not other two kinds of alliinase, such as alliin lyase 1 precursor (alliinase-1 or cysteine sulphoxide lyase 1) and alliin lyase 2 precursor (alliinase-2 or cysteine sulphoxide lyase 2) among EST data, which had been constructed from Chinese garlic species. Meanwhile, EST data, which had been constructed from Korean garlic species, contained three kinds of alliinase mentioned above. Another interesting point to be addressed here is the tissue-specific difference between cysteine synthase gene expression and alliinase gene expression. Our garlic EST data show that cysteine synthase family genes are expressed in both leaf and stem tissues, while alliinase family genes are expressed only in stem tissue (Additional file 1).

## Conclusion

In summary, the GarlicESTdb is the first incorporated online information database of garlic EST sequences that can be freely accessed and downloaded. Our garlic EST database is not only uniquely comprehensive world-wide garlic EST database, but also the most useful one which includes sufficient information of genes that are representative to the characteristics of garlic in health-giving aspects, and which also, includes important information in investigating the properties of garlic genes in molecular level and gene function level. Moreover, it is a tool for information retrieval, visualization, and management. Consequently, the development of the GarlicESTdb will provide a crucial contribution to biologists for data-mining and more efficient experimental studies.

## Availability and requirements

The GarlicESTdb is freely available at . Curation service requires free user registration because users need a unique session. All questions, comments and requests may be sent by email to todaewon@kribb.re.kr.

## Competing interests

The authors declare that they have no competing interests.

## Authors' contributions

DK designed the project and prepared the manuscript. TJ, SN and DK designed the algorithm, carried out the majority of the analyses and all web-programming, and constructed database system. HK participated in the pre-processing of the raw EST sequences and performed the feasibility test of the tool. AK and two SCs assisted in the preparation of the EST data sets (sample collection, cDNA library construction and sequencing). HP served as the principal investigator of the project. All authors including RK have contributed to the writing of the manuscript and the construction of database, and have read and approved the final submitted version.

## Supplementary Material

Additional file 1 Garlic cysteine synthase and alliinase family. Click here for file

## References

[B1] Wolfsberg TG, Landsman D (1997). A comparison of expressed sequence tags (ESTs) to human genomic sequences. Nucleic Acids Res.

[B2] Yeo G, Holste D, Kreiman G, Burge CB (2004). Variation in alternative splicing across human tissues. Genome Biol.

[B3] Xu Q, Modrek B, Lee C (2002). Genome-wide detection of tissue-specific alternative splicing in the human transcriptome. Nucleic Acids Res.

[B4] Yu JK, Dake TM, Singh S, Benscher D, Li W, Gill B, Sorrells ME (2004). Development and mapping of EST-derived simple sequence repeat markers for hexaploid wheat. Genome.

[B5] Loridon K, McPhee K, Morin J, Dubreuil P, Pilet-Nayel ML, Aubert G, Rameau C, Baranger A, Coyne C, Lejeune-Henaut I (2005). Microsatellite marker polymorphism and mapping in pea (Pisum sativum L.). Theor Appl Genet.

[B6] Kumar CG, LeDuc R, Gong G, Roinishivili L, Lewin HA, Liu L (2004). ESTIMA, a tool for EST management in a multi-project environment. BMC Bioinformatics.

[B7] Hotz-Wagenblatt A, Hankeln T, Ernst P, Glatting KH, Schmidt ER, Suhai S (2003). ESTAnnotator: A tool for high throughput EST annotation. Nucleic Acids Res.

[B8] Nelson RT, Grant D, Shoemaker RC (2005). ESTminer: a suite of programs for gene and allele identification. Bioinformatics.

[B9] Ewing B, Hillier L, Wendl MC, Green P (1998). Base-calling of automated sequencer traces using phred. I. Accuracy assessment. Genome Res.

[B10] Jurka J, Kapitonov VV, Pavlicek A, Klonowski P, Kohany O, Walichiewicz J (2005). Repbase Update, a database of eukaryotic repetitive elements. Cytogenet Genome Res.

[B11] Pertea G, Huang X, Liang F, Antonescu V, Sultana R, Karamycheva S, Lee Y, White J, Cheung F, Parvizi B (2003). TIGR Gene Indices clustering tools (TGICL): a software system for fast clustering of large EST datasets. Bioinformatics.

[B12] Huang X, Madan A (1999). CAP3: A DNA sequence assembly program. Genome Res.

[B13] Benson G (1999). Tandem repeats finder: a program to analyze DNA sequences. Nucleic Acids Res.

[B14] Barker G, Batley J, H OS, Edwards KJ, Edwards D (2003). Redundancy based detection of sequence polymorphisms in expressed sequence tag data using autoSNP. Bioinformatics.

[B15] Jones MG, Hughes J, Tregova A, Milne J, Tomsett AB, Collin HA (2004). Biosynthesis of the flavour precursors of onion and garlic. J Exp Bot.

[B16] Jones MG, Collin HA, Tregova A, Trueman L, Brown L, Corsstick R, Hughes J, Milne J, Wilkinson MC, Tomsett AB, Thomas B (2007). The Biochemical and Physiological Genesis of Alliin in Garlic. Medicinal and Aromatic Plant Science and Biotechnology.

